# Reliability of simulation-based assessment for practicing physicians: performance is context-specific

**DOI:** 10.1186/s12909-021-02617-8

**Published:** 2021-04-12

**Authors:** Elizabeth Sinz, Arna Banerjee, Randolph Steadman, Matthew S. Shotwell, Jason Slagle, William R. McIvor, Laurence Torsher, Amanda Burden, Jeffrey B. Cooper, Samuel DeMaria, Adam I. Levine, Christine Park, David M. Gaba, Matthew B. Weinger, John R. Boulet

**Affiliations:** 1 Penn State University College of Medicine, Hershey, PA 17033 USA; 2 Vanderbilt University School of Medicine, Nashville, TN USA; 3 Houston Methodist, Houston, TX USA; 4 Vanderbilt University, Nashville, TN USA; 5 Center for Research and Innovation in Systems Safety, Vanderbilt University, Nashville, TN USA; 6 WISER Simulation Center, University of Pittsburgh School of Medicine, Pittsburgh, PA USA; 7 Mayo Clinic, Rochester, MN USA; 8 Cooper Medical School of Rowan University, Cooper University Hospital, Camden, NJ USA; 9 Harvard Medical School, Massachusetts General Hospital, Senior Fellow, Center for Medical Simulation, Boston, MA USA; 10 Icahn School of Medicine at the Mt Sinai Medical Center, New York, NY USA; 11 Department of Medical Education, Simulation and Integrative Learning Institute, University of Illinois College of Medicine, Chicago, IL USA; 12 Stanford University and Staff Physician and Founder/Co-Director Simulation Center, VA Palo Alto, Palo Alto, CA USA; 13 Center for Research and Innovation in Systems Safety (CRISS), Institute for Medicine and Public Health, Vanderbilt University Medical Center, Vanderbilt University School of Medicine, Nashville, TN USA; 14 Foundation for the Advancement of International Medical Education and Research (FAIMER), Philadelphia, PA USA

**Keywords:** Generalisability, Program evaluation, Simulation, Assessment, Practicing physicians, Feedback, Competency, Continuing medical education

## Abstract

**Introduction:**

Even physicians who routinely work in complex, dynamic practices may be unprepared to optimally manage challenging critical events. High-fidelity simulation can realistically mimic critical clinically relevant events, however the reliability and validity of simulation-based assessment scores for practicing physicians has not been established.

**Methods:**

Standardised complex simulation scenarios were developed and administered to board-certified, practicing anesthesiologists who volunteered to participate in an assessment study during formative maintenance of certification activities. A subset of the study population agreed to participate as the primary responder in a second scenario for this study. The physicians were assessed independently by trained raters on both teamwork/behavioural and technical performance measures. Analysis using Generalisability and Decision studies were completed for the two scenarios with two raters.

**Results:**

The behavioural score was not more reliable than the technical score. With two raters > 20 scenarios would be required to achieve a reliability estimate of 0.7. Increasing the number of raters for a given scenario would have little effect on reliability.

**Conclusions:**

The performance of practicing physicians on simulated critical events may be highly context-specific. Realistic simulation-based assessment for practicing physicians is resource-intensive and may be best-suited for individualized formative feedback. More importantly, aggregate data from a population of participants may have an even higher impact if used to identify skill or knowledge gaps to be addressed by training programs and inform continuing education improvements across the profession.

## Background

Despite advanced training, physicians working in complex, dynamic practices may not be prepared to optimally manage uncommon, challenging critical events. High fidelity simulation is increasingly used across all levels of training and is effective for improving the performance of individual physicians in a manner that is highly relevant to actual clinical activities [1]. We recently demonstrated that simulation-based assessment using complex scenarios requiring critical thinking and crisis resource management skills can be developed and administered in a standardised way at multiple sites [2]. Nevertheless, issues concerning the reliability and validity of simulation-based performance assessment scores for practicing physicians, both individually and as members of a team, still remain.

Performance assessments are generally constructed to measure key skills across multiple situations. However, due to context-specificity, performance in one particular clinical context may not be predictive of performance in another [3–5]. As such, reliable estimates of ability require many performance samples. Although assessment using computer-based simulations [6] and standardised patients [7, 8] has been studied over decades, relatively little work has explored the generalisability of assessment scores for specific measures of ability using manikin-based simulation. Moreover, most prior research has focused on residents, not practicing physicians, and has involved relatively short scenarios that, arguably, lack the fidelity of real-world clinical events [9, 10]. The evaluation of the generalisability, or reliability, of team management skills has yet to be thoroughly investigated.

Many high-stakes performance assessments focus on straightforward interactions or problems. While simple, uncomplicated, scenarios may be appropriate for some tests, they are not likely appropriate for assessing the ability of practicing anesthesiologists to manage critically ill patients. To assess practicing physicians, realistic scenarios that require integration of multiple skills, such as the patient and team management simulations described here, are needed. Unfortunately, these types of scenarios are typically very context specific. This makes it challenging to administer a sufficient number of scenarios to yield reliable and fair estimates of an individual clinician’s abilities and still be practical in terms of available resources.

If simulation scenarios and associated tasks are indeed reflective of actual practice, and assessment scores discriminate along the ability continuum, areas where patient care is suboptimal can be identified. Simulated life-threatening situations that require time-sensitive interventions can reveal individual performance gaps and direct further training [11]. In addition, an ongoing population-wide evaluation of the abilities of physician groups could provide feedback to training programs and departmental leadership, and help inform changes to continuing medical education curricula. Through deeper analysis of a subset of data from the study by Weinger et al. [2], we begin to address whether simulation-based assessment, employing manikins in high-fidelity clinical scenarios provide reliable estimates of the technical and behavioural performance of practicing anesthesiologists.

## Methods

### Scenarios

We developed a set of standardised scenarios, endorsed by subject matter experts and scripted to include acceptable timing and actions of salient events. Scenarios included standardised supporting materials (e.g., patient encounter records, images or laboratory results) and required interactions with a variety of health-care team members (e.g., surgeons, nurses, other anesthesiologists). Study scenarios represented a range of clinical settings and crisis events that practicing anesthesiologists are expected to be able to manage, including local anesthetic toxicity (LAST), malignant hyperthermia (MH), and acute occult intraoperative hemorrhage (Hemorrhage). Cases were specifically designed to measure diagnostic, clinical management, and teamwork skills. A summary of the scenarios is outlined in Table 1 and full details about case development and evaluation can be found elsewhere [2, 12].

**Table 1:** Perioperative Critical Event Scenarios

Scenario (Task)	Setting	Procedure	Critical Patient Issue	Critical Communication
LAST	Outpatient procedural suite	Dilation and curettage with a paracervical block	Local anesthetic systemic toxicity leading to hemodynamic collapse	Obstetrician Sedation Nurse 2nd Anesthesiologist
MH	Post-anaesthesia recovery unit (PACU)	Endoscopic retrograde cholangiopancreatography (ERCP)	Malignant hyperthermia presenting in the post anesthesia care unit	Gastroenterologist Recovery Room Nurse Respiratory Therapist 2nd Anesthesiologist
Hemorrhage	Operating Room (OR)	Pelvic laparoscopic surgery	Occult retroperitoneal hemorrhage secondary to an iatrogenic injury leading to hemodynamic instability and shock	Surgeon OR circulating nurse 2nd anesthesiologist

### Sample population

This report focuses on a small subset of participants within a larger study that included 263 practicing anesthesiologists who provided written consent to have their performance assessed at one of eight study sites during Maintenance of Certification in Anesthesiology courses. All study volunteers performed two scenarios, once as the anesthesiologist primarily in charge (termed the ‘Hot Seat’) and once as the ‘First Responder’ who helped the ‘Hot Seat”. While most of the 263 participants only managed a single scenario as the ‘Hot Seat’, a subset of eighteen subjects from four of the study sites volunteered to be assessed in the ‘Hot Seat’ role in a second scenario for this study; ten were primary responders in the LAST and MH cases and eight were primary responders in the LAST and Hemorrhage cases.

### Measures

Two sets of performance measures were collected for each scenario: non-technical skills (Behavioural) and applied medical knowledge/management skills (Technical). Behavioural skills were assessed via a Behavioural Anchored Rating Scale (BARS), a 9-point scale (1–3 = poor; 7–9 = excellent) along 4 dimensions (Vigilance/Situation Awareness; Decision-Making; Communication; Teamwork). A BARS total score was derived from the mean of the 4 dimension scores [13]. Global ratings of technical performance were assessed on a similar 9-point scale [2].

### Raters/ rating process

All ratings were determined by board certified anesthesiologists with at least 3 years of post-certification clinical practice and experience with high-fidelity simulation. All raters participated in a 2-day, in-person training session and were calibrated via an intensive set of certification procedures described in detail elsewhere [2]. Nine academic anesthesiologists, previously unaffiliated with the study, were selected as potential raters. A panel of project team members established consensus ratings on 24 exemplar study videos to be used as “gold standards” for training and assessment. During initial group in-person training sessions and throughout one-on-one training sessions, we provided video exemplars of each performance dimension. This gave video rater trainees a frame of reference for each performance level for each study scenario. Rater training included education about common errors that can be made in the rating process (e.g., halo and pitchfork effects, fixation, etc.) and these were identified if a rater appeared to make such errors throughout their training.

Project team members mentored the raters, providing one-on-one guidance, first in person and then via videoconference. Rater calibration was assessed regularly during training until the raters’ CPE ratings matched the consensus ratings exactly, their BARS scores were no more than 1-point from the consensus rating, and their performance ratings were within the same preliminary “bin” for the holistic behavioural and technical ratings. Seven raters successfully completed the training and were able to rate performances in all four scenarios consistently.

These seven raters, blinded to the encounter site, viewed and rated recordings of scenarios via an online system that allowed for efficient review and replay. Videos were assigned to the raters in blocks using a randomization tool with a second randomization to prevent viewing performances by the same participant in consecutive videos. Each performance was independently rated by two certified raters who were compensated for their time. Rater performance was re-assessed periodically and there was no drift of individual scores over time.

### Analyses

We generated descriptive statistics of scenario difficulty and rater stringency for behavioural and technical ratings. Relationships between the ratings provided by the two raters were quantified using Pearson correlations. The relationships between the performances on the two scenarios for each participant, based on the average of the two rater’s scores, were also quantified using Pearson correlations.

As part of a Generalisability (G) study, variance components were calculated and analysed to explore sources of measurement error in the scores [14, 15]. This was done separately for each of the two outcome measures. As part of the rating assignment, ten participants were administered the same two scenarios (LAST/MH). Each of these two scenarios, henceforth referred to as ‘Task’, was independently scored by two raters. This resulted in a fully crossed Person (P) by Task (T) by Rater (R) design. Eight other participants performed the LAST/Hemorrhage combination of scenarios that were rated independently by the same two raters, resulting in another fully crossed P by T by R study.

Variance components from the two fully crossed experiments were calculated based on the two outcome measures. The variance components were used to estimate the reliability of the scores. Decision (D) studies were conducted to explore how changes to the facets (number of raters, number of stations) would impact measurement precision. Assuming the scenarios are representative of the domain of interest, and assuming the raters were representative of those who would evaluate the practicing anesthesiologists, the D studies also allowed us to estimate the reliability of the scores for different numbers of raters and scenarios.

## Results

There were eight female and ten male participants. The average age was 40.7 years (SD = 6.7). The participants, on average, had 7.8 years (range, 3–21) experience as practicing anesthesiologists (post-residency training). They supervised, on average, 91 anesthetics per month (range 0–255; SD = 85) and personally performed 41 (range 0–100; SD = 34.0). Half of the participants (*n* = 9) had no previous simulation experience. The majority (*n* = 11, 61.1%) practised in a community setting vs. an academic one.

Table 2 provides the mean ratings by scenario and rater, stratified by the scenario pairing (LAST/MH; LAST/Hemorrhage). For the LAST/MH pairing (pair 1), performance on the LAST scenario tended to be rated higher. For the LAST/Hemorrhage pairing (pair 2), the Hemorrhage scenario performance was rated higher. For both scenario pairs, rater 1 tended, on average, to provide higher scores.

**Table 2:** Mean Scores for Behavioural and Technical Performance by Scenario and Rater

	Behavioural		Technical	
Pair 1 (*n* = 10)	R1	R2	R1	R2
LAST	6.4 (2.0)	5.6 (2.3)	5.9 (2.1)	5.3 (1.8)
MH	5.5 (1.9)	5.6 (2.2)	5.3 (2.2)	5.1 (2.2)
Pair 2 (*n* = 8)				
LAST	3.7 (1.9)	2.8 (1.1)	4.5 (1.7)	2.6 (1.3)
HEMORRHAGE	6.7 (2.3)	5.2 (2.3)	6.5 (2.5)	4.6 (2.5)

Independent of the scenario being rated, the correlations between *rater scores* were 0.69 and 0.57 for the Behavioural and Technical measures, respectively. Averaging over raters, the correlations between scenario performances for individual participants for the 2 outcome measures were 0.21 and 0.22, respectively.

The estimated variance components for the Generalisability studies are presented in Tables 3 and 4. While there are 2 outcome measures, only the results for Behavioural measure are described in detail. This is done to illustrate the interpretation of variance components and the associated generalisability (reliability) estimates for the scores. The interpretation of the variance components for the Technical measure is similar.

**Table 3:** Variance Components for LAST/MH Pairing

	Behavioural		Technical	
Source		%		%
Person (P)	1.10	23.8	1.01	23.3
Task (T)	0	0	0	0
Rater (R)	0	0	0	0
Person x Task	2.14	46.2	1.93	44.6
Person x Rater	0.42	9.1	0.60	13.9
Task x Rater	0.16	3.4	0	0
Error	0.81	17.6	0.79	18.2
G (R = 2, T = 2)	0.43		0.41	
G (R = 1, T = 10)	0.61		0.54	
G (R = 2, T = 20)	0.76		0.71

**Table 4:** Variance Components for LAST/Hemorrhage Pairing

	Behavioural		Technical	
Source		%		%
Person (P)	0.69	8.8	0.50	6.4
Task (T)	3.51	44.8	1.86	23.8
Rater (R)	0.51	6.5	1.70	21.7
Person x Task	1.21	15.5	1.14	14.6
Person x Rater	0.93	11.9	0.49	6.3
Task x Rater	0	0	0	0
Error	0.99	12.6	2.14	27.4
G (R = 2, T = 2)	0.34		0.27	
G (R = 1, T = 10)	0.37		0.43	
G (R = 2, T = 20)	0.56		0.58	

### LAST/MH scenario pairing (Behavioural)

#### Generalisability (G) study

The person (participant) variance component is an estimate of the variance across participants of their mean scores. Ideally, most of the variance should be here, indicating that individual abilities account for differences in observed scores. The other main effect of variance components include task (scenario) and rater. The task component is the estimated variance of scenario mean score. Since the estimate is 0 (see Table 3), the two tasks did not vary much with respect to average difficulty. Mean performance for the 2 simulation scenarios was 5.9 and 5.6. The rater component is the variance of the rater mean scores. The zero estimate indicates that the raters did not vary in terms of average stringency. Overall, raters differed about as much in average stringency as scenarios differ in average difficulty. The largest interaction variance component was person by task. The magnitude of this component suggests that there were considerably different rank orderings of participant mean scores for each of the two simulation scenarios. The non-zero person by rater component suggests that the raters did not rank order the persons similarly. The relatively small rater by scenario component indicates that the raters rank ordered the difficulty of the simulation scenarios similarly. The final variance component is the residual variance which includes triple order interactions and all other unexplained sources of variation. Ideally, this value should be as small.

#### Decision (D) studies

The G Study (above) is used to derive estimates of the variance components associated with the universe of admissible observations. Decision studies can use these estimates to explore efficient measurement procedures. For this investigation, we chose to generalise participants’ ratings based on the two scenarios and two raters to participants’ ratings for a universe of generalization that includes many other scenarios (from the anaesthesiology domain) and many other raters (of similar experience and with similar training).

The generalisability coefficients for various possible combinations of raters and scenarios are presented at the bottom of Table 3. Based on the magnitude of the variance components, it is clear that the reliability of the simulation scores is more dependent on the number of scenarios than the number of raters. A model with 10 scenarios and 1 randomly selected rater for each would yield a reliability estimate of 0.61. Doubling the number of scenarios (scenarios = 20) and including 2 raters (per scenario) would yield a reliability estimate of 0.76.

### LAST/ hemorrhage scenario (Behavioural)

#### G study

The variance components for this pairing of scenarios (Table 4) are somewhat different from those reported for the LAST/MH pairing. While there is still appreciable variance attributable to person by task, indicating that there are considerably different rank orderings of participants’ mean scores for each of the two simulation scenarios, nearly 45% of the variance is attributable to scenario. This suggests that these two scenarios are not of equal difficulty. The average Behaviour score for the LAST scenario was 3.2; the average score for Hemorrhage scenario was 5.9.

#### D studies

The estimated score reliability for a model with two scenarios and two raters (per scenario) is 0.34. Increasing the number of scenarios to 20 would yield an estimated generalisability coefficient of 0.56.

### Comparison of reliability estimates

An inspection of the variance components, across outcome measures, and between scenario pairings, yields several important findings. First, regardless of the measured construct, a substantial number of scenarios (> 20, with 2 raters) would be needed to achieve a reliability estimate of 0.70, a value considered minimally acceptable for most assessments [16]. The average G coefficients for Behavioural and Technical scores, based on two scenarios and two raters, were 0.39 and 0.34, respectively. Second, both the scenario pairing and the choice of outcome measure had some impact on the generalisability of the scores. Third, the Behavioural score was, in general, no more reliable than the Technical one. Finally, while more pronounced for the LAST/MH pairing, there was a large amount of variance attributable to Person x Task (Scenario).

## Discussion

We found that the performance of practicing specialist physicians on complex, realistic simulated critical event scenarios involving teamwork is highly context-specific. Context-specificity has been identified for technical and communication skills in computer-based simulations for medical students in the 1980’s [5, 6], and assessment using standardised patients in 2004 [8]. Variation due to task sampling (context specificity attributable to the content of the scenario) is known to affect the validity and reliability (generalisability) of scores [17, 18]. However, measurement properties of assessment scores have not been well characterized for the population of practicing anesthesiologists we studied, nor have they been well evaluated for the types of critical event scenarios we modeled. The simulation cases used in this study were constructed to reflect the timing of events in actual clinical practice in scenarios that require both technical and behavioural expertise to effectively manage the patient’s condition. These cases were presented with as much realism as is achievable with current simulation techniques. Scenarios were developed to accurately reflect the types of challenges faced by practicing anesthesiologists in real-world, emergency situations where the correct answer is not clear and the outcome is not predetermined. Thus, we believe that the performances elicited were likely a fair reflection of how the subjects would have acted in real situation, although that cannot be know with certainty. Yet, even with some reservations about drawing strong conclusions from this data about the reliability of an assessment using this approach, we were surprised to find that the performance of physicians in one of two critical event simulation scenarios often did not predict their performance in the other. This was true for both behavioural and technical ability.

Previous studies have investigated the psychometric properties (reliability, validity) of scores from standardised patient encounters as well as other simulation-based assessments [19–21]. While reliable and valid scores and associated high stakes competency decisions can be obtained, these decisions demand broad sampling of the domain and effective rater training [22]. High-stakes applications, such as the introduction of objective structured clinical examinations (OSCE) into the primary board certification of anesthesiologists [23], require an evaluation of the sources and magnitude of measurement error to determine the number of scenarios needed to obtain sufficiently precise estimates of ability. The American Board of Anesthesiology (ABA) recently introduced OSCEs to assess two domains that “may be difficult to evaluate in written or oral exams - communication and professionalism and technical skills related to patient care” [24]. Those examinations are comprised of seven stations. Other certification bodies, including the Royal College of Physicians and Surgeons of Canada, also realize the unique ability of simulation-based assessment to evaluate domains not covered with traditional assessment techniques [25]. However, the types of assessment encounters administered can be highly context specific, that is, because of the nature of the management task, the skills measured in one patient management problem may not generalise to another. This indicates that numerous performance samples are needed to get sufficiently reliable ability estimates.

Despite the validity advantages of assessment based on real or realistic clinical encounters, the inconsistent performance by trainees on different cases, and the variability of assessment judgements has necessitated the use of simpler or focused cases that typically lead to results that are similar to a cheaper and easier test such as a multiple choice written exam [5]. Lengthy and expensive examinations are not considered valuable to practicing clinicians, and as Van Der Vleutin posits, “Assessment not accepted by staff or students will not survive.” But individual scores are not the only, nor even the most important use for clinical assessment. Test results can be used for individual reflection, feedback for instructors, and quality monitoring of training programs. Moreover, the input of multiple assessors may capture different meaningful aspects of highly complex and nuanced performance within the same case or across a range of cases [26]. While inconsistent and unreliable scoring may be problematic for certification examinations, these cases may be highly valuable for participant growth and development.

The D study, although limited because each participant was only evaluated in three different scenarios in two pairs, suggests that greater than 20 scenarios would be required to achieve a reliability of 0.8 (desirable for high-stakes assessments [16]). Controlling for numbers of scenarios and raters, the estimated generalisability coefficients from our study were lower than those reported elsewhere [27, 28]. While the scenarios were modeled to present management challenges that all practicing, board certified, anesthesiologists should be able to handle, we found that some participants could perform well on one scenario and do poorly on the next. A similar observation was made in a recent analysis of anaesthesiology residents who were scored on four simulation scenarios [29]. In our analysis, this variation was seen in both technical and behavioural performance. The scenarios were developed to elicit nuanced performances that may have been more content specific because clear-cut management expectations were accompanied by ambiguous, real world interactions with others embedded in various provider roles within the scenario. This result highlights the challenge of developing content-valid and practice-relevant simulation-based performance assessments for practicing physicians, especially if these are to be used for summative purposes.

For most performance-based assessments, the variance attributable to the task, and associated interactions, outweighs that associated with the rater [30]. While variance attributable to the rater was less than that attributed to the task (scenario), it was not zero for the second pair of scenarios. Even though rater training was quite stringent, individual evaluators still varied with respect to how they used the scoring rubrics. Also, with longer scenarios, the raters had to aggregate holistic judgments over time, potentially leading to more variation between raters. Future studies could explore these potentially biasing effects by collecting performance ratings over time and comparing these with overall judgments. As it stands, at least for the types of complex scenarios modeled in our investigation, the ability estimates of the practitioners were highly dependent on the choice of scenarios and, to a lesser extent, the choice of raters.

To improve reliability, the problem of context specificity can be addressed in a number of ways, including shortening the scenarios (to allow for the collection of more performance samples) and making scenario content more generic. A study of junior anaesthesiology trainees that used a behaviourally anchored ratings system to score seven, 15-min scenarios achieved a generalisability coefficient of 0.81 [11]. However, shortening the scenarios, while increasing sample sizes, could have a negative impact on validity. One of the strengths of utilizing longer scenarios is that they more accurately represent the clinical environment, thus allowing for the assessment of patient management strategies over a realistic, evolving, event.

We intentionally scored behavioural and technical skills separately, hypothesizing that behavioural skills would be less content specific than technical skills. Our results did not support this hypothesis; behavioural performance was as scenario-specific as technical performance. While one might expect that behavioural skills would be more generalizable across different patient encounters, communication skills have been found to be domain specific in other work-based assessments^26^. To overcome this confounder, typical standardised patient scenarios that measure doctor-patient communication are focused and graded using a process-based checklist [31], to provide reliable assessment of particular skills. Our scenarios included communication with various providers, including a first-responder anesthesiologist, other physicians, and various healthcare professionals and this likely affected the generalisability of communication skills measurement. It is likely that for actual critical events the context and criticality of the patient presentation, as well as the particular person, or persons present have a significant effect on both the technical and non-technical skills.

Our results suggest that a robust simulation-based high-stakes performance assessment for practicing anesthesiologists would be challenging and, perhaps, impractical. We hesitate to make such a firm conclusion because of the limited number of samples for each subject in this analysis. Regardless of the practicality of simulation for high-stakes assessment, formative assessment of individual performance in these kinds of longer, more complex, critical event scenarios still has considerable value for individuals as well as for learning how clinicians perform in general. Numerous studies have shown that simulation-based medical education fosters self-reflection and identification of performance gaps [32–34]. As part of ongoing professional improvement, providing feedback to individual physicians about their performance on the management of specific clinical emergencies is likely to have a positive impact on the quality of their subsequent patients’ care. Additionally, standardised technical and behavioural learner-specific feedback would likely have a greater impact on the learner’s awareness of their knowledge and performance gaps for a particular event than self-assessment. This use of simulation could be initiated using the scenarios and assessment tools we have developed. Objective, specific feedback should have a positive long-term impact on the quality of patient care delivered by individuals who participate in these formative, simulation-based assessments [35].

Although there have been numerous changes in undergraduate medical education and residency training guidelines, “graduate medical education (GME) lacks a data-driven feedback system to evaluate how residency-level competencies translate into successful independent practices...” [36]. Simulation-based performance data from practicing clinicians could be aggregated to inform modifications in educational and training programs to address specific performance deficiencies across specialties. The impact of this approach for the profession and our patients might actually be greater than administering high-stakes summative examinations because the goal would be to raise the performance of the entire profession rather than to identify and restrict the low performers from practicing.

Our study had a number of limitations, most importantly the small group of participants who agreed to being studied as the primary provider in two scenarios. To the extent that these participants are not representative of practicing anesthesiologists as a whole, the generalisability of our findings could be questioned. A larger-scale study, where participants are required to manage more scenarios, would better quantify the effect of task sampling on the reliability of the scores. Although the order of the cases was not randomised specifically for this subset, it was also not prescribed, and neither of the two cases could be the first case of the day. Further, our study was limited to two independent ratings of each scenario. While rater effects should tend to cancel out with sufficient numbers of scenarios and raters, we were not able to adequately investigate this. For future studies specifically designed to assess the numbers of scenarios and raters needed to achieve adequate reliability for high-stakes assessment, it would be appropriate to incorporate a design where more participants managed a larger number of encounters and with more raters.

Second, the study was embedded within a required formative educational experience for board-certified anesthesiologists [33] and this affected the design of the scenarios, which were found to have some differences in difficulty. Although this may be attributable to the clinical problem being managed, it may also have been a reflection of a scenario that was not optimally designed or administered and hence was more difficult for the participants to interpret and manage. For example, the LAST case may have been more challenging than anticipated due to the unrealistic portrayal of seizures by manikins. Since the cases were primarily designed for formative education, the content, timing, and delivery may have been affected. Thus our results may not fully generalise to a high-stakes assessment setting where both individual factors (e.g. motivation) and environmental factors could be quite different.

## Conclusions

Despite the limitations, our study showed that performance in complex manikin-based simulation encounters is context specific. The administration of a larger number of scenarios would yield a more reliable assessment of an individual’s clinical performance but would be logistically challenging and increase the costs. However, the use of relatively few scenarios still allows for the provision of individual, case-specific, feedback to clinicians. Given the rarity of some clinical presentations, the performance data, in aggregate, could also inform quality improvement initiatives, including focused training programs and educational activities.

## Data Availability

Not applicable.

## Abbreviations

ABA: American board of anesthesiology
BARS: Behavioural anchored rating scale
GME: Graduate medical education
LAST: Local anaesthetic toxicity
MH: Malignant hyperthermia
OSCE: Objective structured clinical examination
